# ShinySOM: graphical SOM-based analysis of single-cell cytometry data

**DOI:** 10.1093/bioinformatics/btaa091

**Published:** 2020-02-12

**Authors:** Miroslav Kratochvíl, David Bednárek, Tomáš Sieger, Karel Fišer, Jiří Vondrášek

**Affiliations:** b1 Institute of Organic Chemistry and Biochemistry AS CR, 166 10 Praha 6, Czech Republic; b2 Department of Software Engineering, MFF, Charles University, 118 00 Prague, Czech Republic; b3 Department of Cybernetics, Faculty of Electrical Engineering, Czech Technical University in Prague, 121 35 Prague 2, Czech Republic; b4 Childhood Leukaemia Investigation Prague (CLIP), 2nd Faculty of Medicine, Charles University and University Hospital Motol, 150 06 Praha 5, Czech Republic

## Abstract

**Summary:**

ShinySOM offers a user-friendly interface for reproducible, high-throughput analysis of high-dimensional flow and mass cytometry data guided by self-organizing maps. The software implements a FlowSOM-style workflow, with improvements in performance, visualizations and data dissection possibilities. The outputs of the analysis include precise statistical information about the dissected samples, and R-compatible metadata useful for the batch processing of large sample volumes.

**Availability and implementation:**

ShinySOM is free and open-source, available online at gitlab.com/exaexa/ShinySOM.

**Supplementary information:**

Supplementary data are available at *Bioinformatics* online.

## 1 Introduction

Flow cytometry enables fast measurement of multiple parameters of individual cells in solution. In recent years, multiple algorithms have emerged for analyzing flow and mass cytometry data. Among them, FlowSOM (van Gassen *et al.*, 2015) has been shown to be one of the best performers for automated unsupervised detection of cell populations (Weber and Robinson, 2016). The combination of self-organizing maps (SOMs) and metaclustering leads to high-precision clustering in near real-time, even on large datasets.

The performance of FlowSOM makes it a suitable basis for constructing interactive data analysis platforms. Here, we introduce ShinySOM, a web-based frontend for FlowSOM-style sample dissection and analysis. ShinySOM exposes the FlowSOM functionality in a graphical interface that is useful both for quick interactive and ad-hoc exploration of datasets, and for running the usual FlowSOM-style pipelines in a way convenient for users not familiar with programming interfaces. In addition, ShinySOM provides improved visualization methods and new implementations of unsupervised analysis algorithms, which lead to a substantial speed-up of the operations and further extend the ability to work with large datasets.

## 2 Analysis workflow and methods

**Fig. 1.**
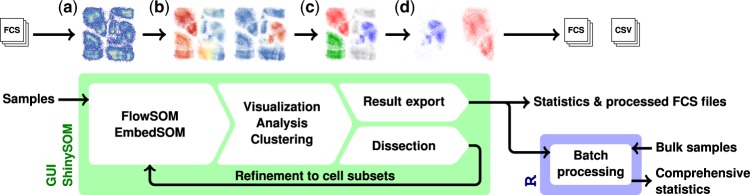
Schema of a typical ShinySOM-backed workflow. Samples are analyzed and dissected in the interactive environment (left box). Example visualizations of the data available at the individual steps are displayed above (a, SOM-based embedding; b, population identification based on marker expressions; c, cluster assignment; d, dissection). Large sample volumes can be subjected to the same analysis as conducted in the GUI by utilizing the exported analysis metadata in a non-interactive environment (right box)

The implemented analysis workflow (illustrated in Fig. 1) follows the general FlowSOM usage guidelines: the user creates a dataset by aggregating several prepared samples, possibly scaling and transforming the marker expressions to match the expectations about data. A SOM is then trained on the dataset to serve as a basis for further analysis.

The SOM is used for multiple purposes: an embedded single-cell view of the samples is computed from the SOM using the fast EmbedSOM algorithm (Kratochvíl *et al.*, 2019). Clustering of the cells is conducted in an interactive dendrogram (Sieger *et al.*, 2017) built from hierarchical metaclustering of the SOM content. Notably, ShinySOM supports the agglomerative clustering with Mahalanobis-average linkage (Fišer *et al.*, 2012) that has been shown to be capable of defining cell populations with the same accuracy as expert cytometrists in a real life setting. After selecting the cell populations, the user may display and analyze the differences between samples and sample groups, and visualize the results of statistical testing of the differences in cell abundance. Finally, the annotated populations may be partitioned into smaller datasets, in order to be explored in more detail.

Analysis and annotation results may be exported either as FCS files compatible with other flow-cytometry software, CSV files with population statistics and as RDS files with analysis metadata useful for other R-based workflows.

The implementation of ShinySOM aims to improve the interactive workflow by exposing new algorithm and analysis methods and minimizing the delays caused by computation:

- The computationally intensive parts of the software were re-implemented in efficient low-level code, reducing the required time approximately 3–10× when compared with the original implementations (see Supplementary Fig. S1 for details).
- The Mahalanobis-average linkage computation (Fišer *et al.*, 2012) was ported to the R package mhca (github.com/tsieger/mhca) and customized to work with FlowSOM-style clustering.
- An idendro-inspired (Sieger *et al.*, 2017) Shiny-compatible tool shinyDendro (github.com/exaexa/shinyDendro) was implemented and integrated into ShinySOM, in order to simplify interactive cluster selection and annotation.

The resulting workflow gives results comparable to FlowSOM, while enabling user-controlled cluster selection in the final stage of analysis (see Supplementary Fig. S2). This approach leverages both the strength of unbiased algorithmic data processing and the flexibility of the curated selection of clusters based on domain expertise. The obtained results compare well to manual analysis (see Supplementary Fig. S3).

All used algorithm parameters and user-generated metadata can be exported along with the main analysis results. This information describes all expert decisions performed during the analysis and allows for reproducible use of the platform. Moreover, the ‘canned’ expert decisions may be used to automate the analysis, further allowing, e.g. the construction of R-based batch-processing pipelines.

## 3 Conclusion

ShinySOM provides a new software alternative for analyzing multidimensional flow and mass cytometry data. Importantly, it contains a suite of fast algorithms in a user-friendly environment capable of generating precise results more quickly than the manual gating-based analyses. Accessible use of unsupervised analysis algorithms helps to overcome the common dimensionality overhead and promotes analysis reproducibility.

The interoperability with the R-based environment provides a link between the expert knowledge gathered using the graphical interfaces and the batch processing required for work with large volumes of samples. A complete cytometry workflow can be constructed with the help of other compatible packages in the R ecosystem (e.g. Catalyst and flowAI; Chevrier *et al.*, 2018; Monaco *et al.*, 2016) that implement data cleaning, compensation and advanced visualization.

## Funding

M.K. and J.V. were supported by ELIXIR CZ (MEYS) [LM2015047]. D.B. was supported by the Charles University Project Progress Q48. T.S. and K.F. were supported by Czech Health Research Council (AZV) [NV18-08-00385]. Funding for open access publication was provided by the Institute of Organic Chemistry and Biochemistry of the CAS [RVO: 61388963].

*Conflict of Interest*: none declared.

## Supplementary Material

btaa091_Supplementary_Data
