# Supplementary material for: ShinySOM: graphical SOM-based analysis of single-cell cytometry data
Source: Bioinformatics. 2020 Feb 12;36(10):3288–9. doi: 10.1093/bioinformatics/btaa091 (PMC7214046; doi:10.1093/bioinformatics/btaa091)
Supplement: btaa091_Supplementary_Data [file btaa091_supplementary_data.pdf]

Supplementary material for

# ShinySOM: Graphical SOM-based analysis of single-cell cytometry data

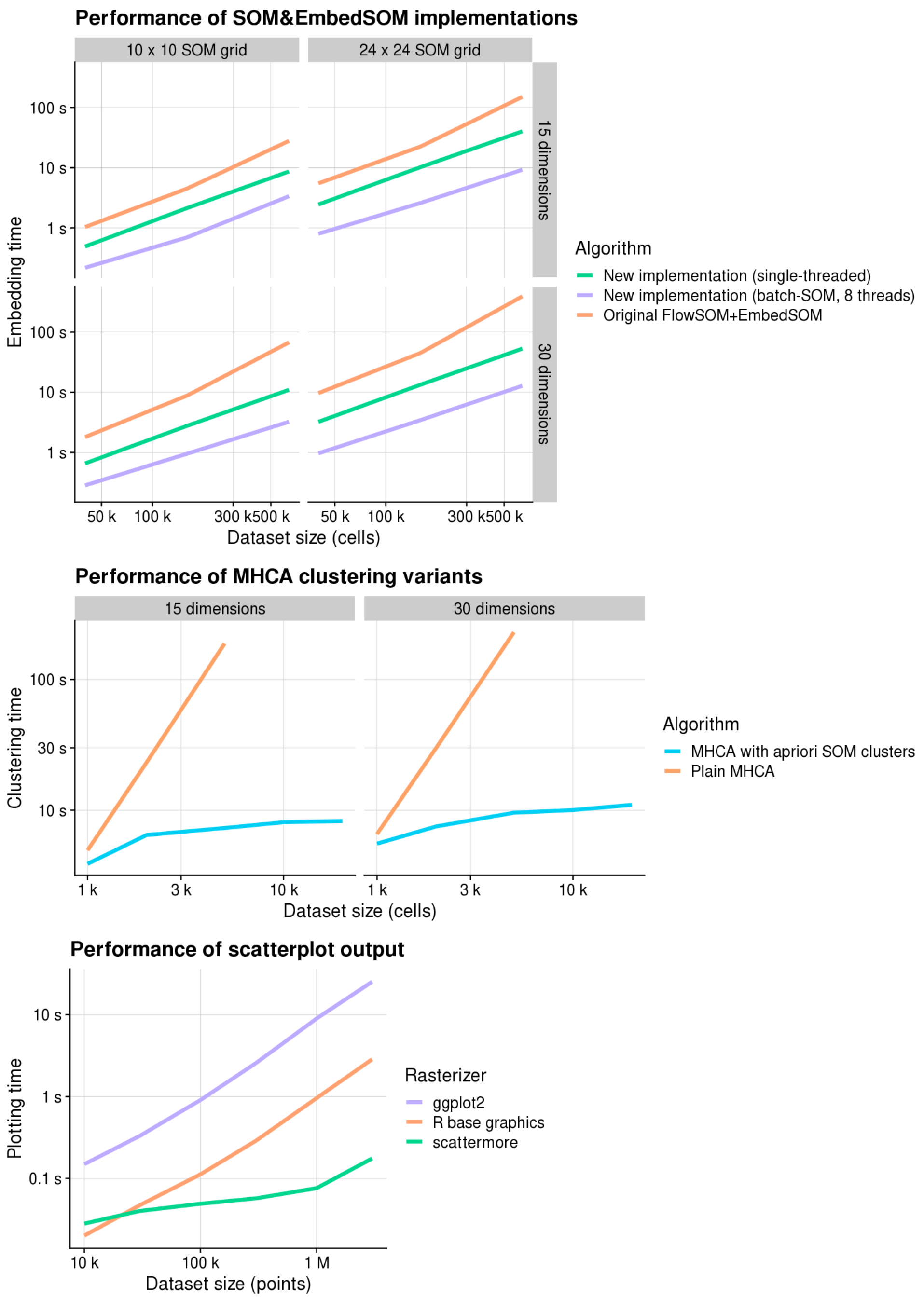


**Fig. S1.** Performance of algorithm re-implementations used in ShinySOM. **Top:** Performance of original EmbedSOM with most computation done by FlowSOM R package, compared to the performance of the SIMD-accelerated single-threaded version, and of the parallelized version thereof. (By design, time complexity of all algorithms scales linearly in number of cells, which makes their performance easily predictable even for large datasets.) **Middle:** Performance of the Mahalanobis linked-average hierarchical clustering algorithm (MHCA) [1] executed on single cells, compared to the same algorithm with apriori clusters prepared from 24×24 SOM (times over 200s were omitted as impractical for interactive environment). **Bottom:** Performance of scatterplot rendering to PNG, the fastest available R rasterizer (Cairo version 1.16) and ggplot2 [4] compared to `scattermore` rasterizer [2] used in ShinySOM. All measurements were conducted on AMD Ryzen 7 2700U CPU with 16GB of RAM running Debian Linux (Bullseye), R version 3.6.1 compiled with gcc version 9.2.1.

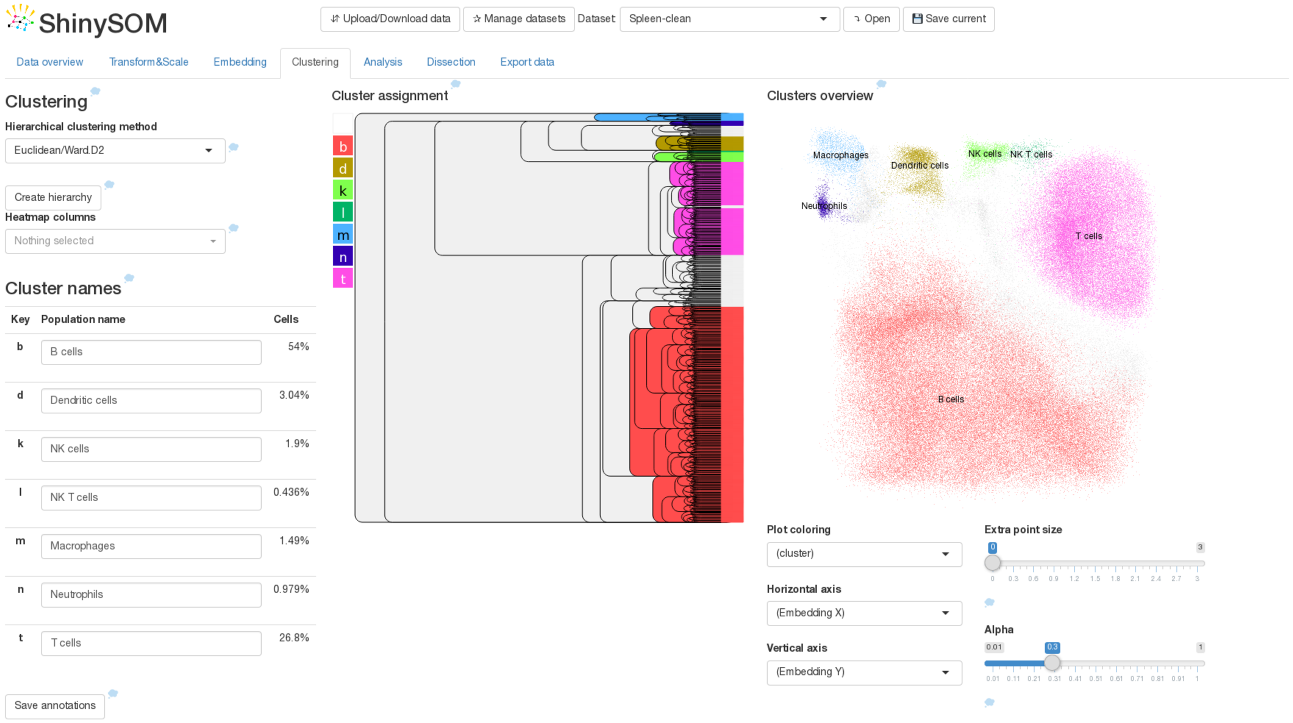


**Fig. S2.** Screenshot of the dendrogram-based cluster assignment tool, running on the dataset 'General immune cell panel on BL6 WT Spleens' (described e.g. by Saeys *et al.* [3]), as viewed in the ShinySOM interface. The dataset is available from FlowRepository under accession ID `FR-FCM-ZZQY`. Additional details about the analysis are available in ShinySOM tutorial at `gitlab.com/exaexa/ShinySOM`.

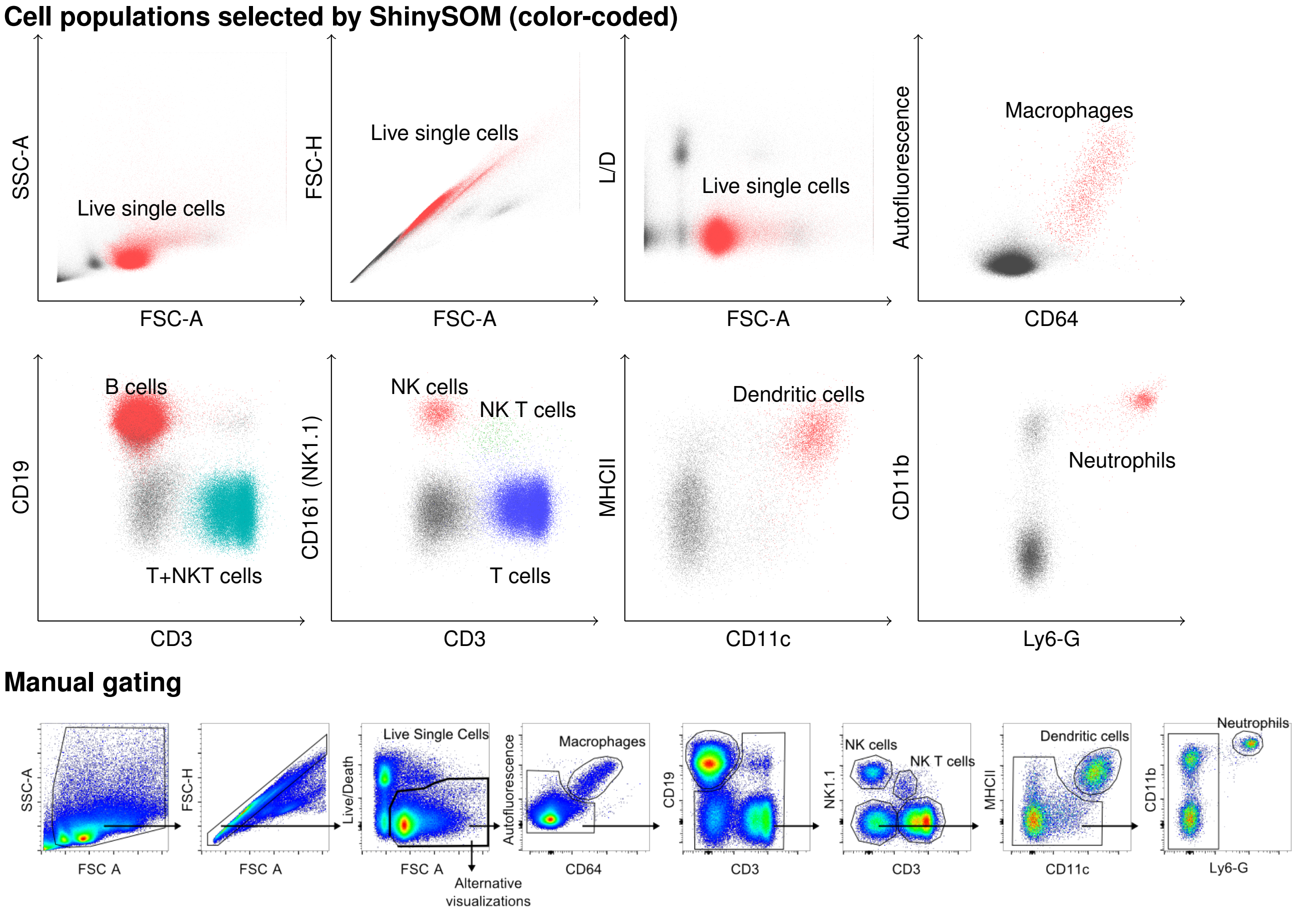


**Fig. S3.** Comparison of ShinySOM output with manual gating. Dot-plot views (above) of the populations selected using the interactive dendrogram tool in the web interface (the data is the same as in Fig. S2) show high correspondence with the usual gating methods (below). Manual gating figure was originally authored by Sofie van Gassen [3], reproduced here from FlowRepository `FR-FCM-ZZQY`.

# References


[1] Fišer, K., Sieger, T., Schumich, A., Wood, B., Irving, J., Mejstříková, E., and Dworzak, M. N. (2012). Detection and monitoring of normal and leukemic cell populations with hierarchical clustering of flow cytometry data. *Cytometry Part A*, **81**(1), 25–34.

[2] Kratochvíl, M. (2019). *scattermore: Scatterplots With More Points*. R package version 0.5.

[3] Saeys, Y., Van Gassen, S., and Lambrecht, B. N. (2016). Computational flow cytometry: helping to make sense of high-dimensional immunology data. *Nature Reviews Immunology*, **16**(7), 449.

[4] Wickham, H. (2016). *ggplot2: Elegant Graphics for Data Analysis*. Springer-Verlag New York.
